# Metformin use and its effect on survival in diabetic patients with advanced non-small cell lung cancer

**DOI:** 10.1186/s12885-016-2658-6

**Published:** 2016-08-12

**Authors:** Oscar Arrieta, Edgar Varela-Santoyo, Enrique Soto-Perez-de-Celis, Roberto Sánchez-Reyes, Martha De la Torre-Vallejo, Saé Muñiz-Hernández, Andrés F. Cardona

**Affiliations:** 1 Thoracic Oncology Unit and Laboratory of Personalized Medicine, Instituto Nacional de Cancerología (INCan), Av. San Fernando 22 Col. Sección XVI, Tlalpan, 14080 Mexico City, Mexico; 2 Cancer Care in the Elderly Clinic, Department of Geriatrics, Instituto Nacional de Ciencias Médicas y Nutrición Salvador Zubirán, Mexico City, Mexico; 3 Clinical and Translational Oncology Group, Institute of Oncology, Clínica del Country, Bogotá, Colombia; 4 Foundation for Clinical and Applied Cancer Research – FICMAC, Bogotá, Colombia

**Keywords:** Diabetes mellitus, Carcinoma, Non-Small-Cell Lung, Metformin, Hypoglycemic agents

## Abstract

**Background:**

Previous population-based studies have demonstrated an association between metformin use and improved survival among diabetic patients with cancer. We sought to analyze the effects of diabetes and its treatment in terms of the survival of patients with lung cancer.

**Methods:**

Overall, 1106 patients with non-small cell lung cancer (94.3 % with stage IV disease) were included. The outcomes were compared between the patients with (*n* = 186) and without diabetes (*n* = 920). The characteristics associated with antidiabetic treatment and proper glycemic control (defined as a mean plasma glucose <130 mg/dL) were examined at diagnosis. The overall survivals (OSs) of the different patient populations were analyzed using Kaplan-Meier curves, and a multivariate Cox proportional hazard model was used to determine the influences of the patient and tumor characteristics on survival.

**Results:**

The OS for the entire population was 18.3 months (95 % CI 16.1-20.4). There was no difference in the OSs of the diabetic and non-diabetic patients (18.5 vs 16.4 months, *p* = 0.62). The diabetic patients taking metformin exhibited a superior OS than did those on other antidiabetic treatments (25.6 vs 13.2 months, *p* = 0.017). Those with proper glycemic control had a better OS than did those without proper glycemic control and the non-diabetics (40.5 vs 13.2 and 18.5 months, respectively, *p* < 0.001). Both the use of metformin (HR 0.53, *p* < 0.0001 and HR 0.57, *p* = 0.017, respectively) and proper glycemic control (HR 0.49, *p* < 0.0001 and HR 0.40, *p* = 0.002, respectively) were significant protective factors in all and only diabetic patients, respectively.

**Conclusions:**

The diabetic patients with proper glycemic control exhibited a better OS than did those without proper glycemic control and even exhibited a better OS than did the patients without diabetes mellitus. Metformin use was independently associated with a better OS.

**Electronic supplementary material:**

The online version of this article (doi:10.1186/s12885-016-2658-6) contains supplementary material, which is available to authorized users.

## Background

In Mexico, more than 95 % of all patients with non-small cell lung cancer (NSCLC) present with advanced-stage disease at the time of diagnosis [1]. Because the prevalence of type 2 diabetes is high both worldwide and in Mexico, it is very important to understand the effects of preexisting diabetes on the survival of patients with advanced NSCLC. The physiopathology of diabetes, the effects of its treatment and the changes in different organ systems observed in diabetic patients might be associated with clinical outcomes. Existing data regarding the effects of diabetes on NSCLC outcomes are conflicting. Although some early studies found longer survival rates in diabetic patients with NSCLC [2], this result was not confirmed in other larger series [3, 4]. Indeed, an elevated fasting glucose level was found to be independently associated with a significantly higher risk of mortality in patients with NSCLC [3].

Among the major hypoglycemic agents that are used for the treatment of diabetes, metformin has received the most attention regarding its potential antineoplastic uses. At the cellular level, metformin has profound effects on the mitochondrial respiration rate and the production of ATP [5]. Metformin affects multiple cellular pathways via the activation of AMP-activated protein kinase (AMPK) by liver kinase 1 (LKB1), which leads to decreases in growth factor signaling and proliferation via the inhibition of mTOR [6].

There is a general lack of information regarding the outcomes of diabetic patients with NSCLC who are treated with different antidiabetic agents, including metformin, and the published data come from large epidemiological databases that are prone to bias [7]. In this study, our objective was to analyze the effects of diabetes and its treatment in terms of the survival of patients with NSCLC.

## Methods

We conducted a retrospective cohort observational study. Consecutive patients diagnosed with NSCLC at the *Instituto Nacional de Cancerología* (INCan) between January 2008 and December 2014 were retrieved from our electronic medical records. The study was approved by INCan’s research committee (REV/05/16). Because the study was deemed as without risk, patient consent was not required. The patient and tumor characteristics, including age, comorbidities, body mass index, stage at diagnosis, tobacco use, wood smoke exposure and mutational analysis results, were obtained. All patients were staged using thoracic and abdominal CT scans, PET scans and MRI imaging of the central nervous system. Previous diagnoses of diabetes, current antidiabetic treatments and glucose measurements for each individual patient were also recorded at the time of the lung cancer diagnosis. The patients were considered to have a diagnosis of diabetes if they either fulfilled the American Diabetes Association (ADA) criteria for diabetes or were being treated with antidiabetic medications prior to diagnosis with NSCLC. The patients were treated according to published guidelines for the treatment of lung cancer [8]. Patients with missing data were excluded from the analysis.

Proper glycemic control was defined by a pre-prandial (fasting) glucose level of 70–130 mg/dL at the time of the lung cancer diagnosis, in accordance with the current guidelines of the ADA [9]. Because most patients lacked a measurement of glycated hemoglobin (HbA1C), we calculated the mean plasma glucose by averaging the patient’s pre-prandial glucose measurements (at least 3). We considered patients with mean plasma glucose levels under 130 mg/dL to be within the proper glycemic control goals and those with a mean plasma glucose level over 130 mg/dL to have improperly controlled diabetes. The overall survival (OS) was calculated from the date of cancer diagnosis to the date of the last visit or death.

The de-identified patient dataset supporting the conclusions of this article is included within the article and its additional supporting file (Additional file 1).

### Statistical analysis

For descriptive purposes, the continuous variables are summarized as arithmetic means with standard deviations (SD) and as medians with ranges. The categorical variables are summarized as the relative frequencies, proportions, and 95 % confidence intervals (95 % CI). The Pearson chi-square test was used to compare the data between the diabetic and non-diabetic patients and between the patients with and without proper glycemic control. The OSs were analyzed using the Kaplan-Meier method, and comparisons between subgroups were performed using the log-rank test or the Breslow test. Statistically significant and borderline significant variables (*p* < 0.1) were included in a multivariate analysis. The Cox proportional hazards model was used to estimate the hazard ratios (HRs) and 95 % CI. The variables that were included in the multivariate analysis were factors that are known to be associated with poor outcomes for patients with NSCLC (i.e., age, ECOG performance status, tumor stage, smoking status, epidermal growth factor receptor (EGFR) mutational status and metastatic disease). The variables of interest in the study (i.e., diagnosis of diabetes, mean plasma glucose and antidiabetic medications) and the variables that were found to be significant in the univariate analysis at the level of *p* < 0.01 were also included in the model. The SPSS software package (version 22.0; SPSS, Chicago, IL, USA) was used for the data analysis. All *p* values presented are two-sided, and *p* values <0.05 were considered statistically significant.

## Results

### Patient characteristics

A total of 1106 patients with diagnoses of NSCLC were identified and considered eligible for the analysis. The median age was 61 years (SD ± 13 years). Most of the patients were male (53 %) and had a history of smoking (58.8 %). ECOG PSs of 0-1 were observed in 75 % of the patients, 68.2 % of the patients had adenocarcinoma histologic diagnosis, and 94.3 % were stage IV (M1a and M1b). Only 417 patients (37.7 %) had undergone EGFR mutation testing, and 152 (36.5 %) of these patients were positive.

Diabetes was present in 186 (16.8 %) of the patients at the time of cancer diagnosis. The characteristics of the diabetic and non-diabetic patients are presented and compared in Table 1. The calculated mean serum glucose was higher in the patients with diabetes than in the non-diabetics (170 mg/dL [±78.5 mg/dL] vs 105 mg/dL [±14.5 mg/dL], *p* < 0.0001). There were no differences in stage, performance status, mutational status, smoking history or gender between the diabetic and non-diabetic patients. Metformin was used as an antidiabetic treatment at the time of diagnosis by 59.7 % of the diabetic patients, and 31.4 % were within the predefined definition of proper glycemic control (Table 2). Eighty-one percent (*n* = 150) of the diabetic patients had completed at least one line of treatment with either chemotherapy or tyrosine kinase inhibitors.

**Table 1:** Comparison of baseline characteristics between diabetic and non-diabetic patients

Characteristic	Non-diabetics (*n* = 920)	Diabetics (*n* = 186)	*p*
	% (n/N)	% (n/N)	
Gender			0.44
Female	47.2 (434/920)	46.2 (86/186)	
Male	52.8 (486/920)	53.8 (100/186)	
Age at diagnosis (mean)			<0.0001
Mean (SD)	51.2 (13.1)	64.5 (10.5)	
Smoking history			0.22
Non-smoker	40.7 (374/920)	44.1 (82/186)	
Smoker	59.3 (546/920)	55.9 (104/186)	
Histology			0.48
Adenocarcinoma	68.3 (628/920)	67.7 (126/186)	
Other	31.7 (292/920)	32.3 (60/186)	
Disease Stage			0.36
II-IIIa	5.5 (51/920)	6.5 (12/186)	
IIIb-IV	94.5 (869/920)	93.5 (174/186)	
ECOG PS			0.31
0–1	74.5(685/920)	78 (145/186)	
2–4	25.5 (235/920)	22 (41/186)	
CNS metastases at diagnosis			0.09
Absent	53.5 (492/920)	47.8 (89/186)	
Present	46.5 (428/920)	52.2 (97/186)	
Basal Glucose			<0.0001
Mean (SD)	105 (14.5)	170 (78.5)	
EGFR mutation (*n* = 417)			0.09
Positive	34.9 (123/352)	44.6 (29/65)	
Negative	65.1 (229/352)	55.4 (36/65)	
KRAS mutation (*n* = 184)			0.60
Positive	16.3. (25/30)	16.7 (5/31)	
Negative	83.7 (128/153)	83.9 (26/31)	

*ECOG PS* Eastern Cooperative Oncology Group Performance Status Score

**Table 2:** Treatment and glycemic control in the diabetic patient population (*N* = 186)

Characteristic	Patients % (n/N)
Metformin	
Yes	59.7 (111/186)
No	40.3 (75/186)
Glibenclamide	
Yes	32.3 (60/186)
No	67.7 (126/186)
Insulin	
Yes	11.3 (21/186)
No	88.7 (165/186)
Other antidiabetic medication	
Yes	5.4 (10/186)
No	94.6 (176/186)
Basal Fasting Glucose	
Mean (± S.D.)	170 (78.5)
Range	74–604
Glycemic Control (*n* = 185)^a^	
Proper (<130 mg/dL^b^)	31.2 (58/185)
Improper (≥130 mg/dL^b^)	68.8 (127/185)

^a^ One patient with missing glucose values

^b^ Calculated mean serum glucose

### Factors associated with survival in the overall cohort

**Fig. 1:**
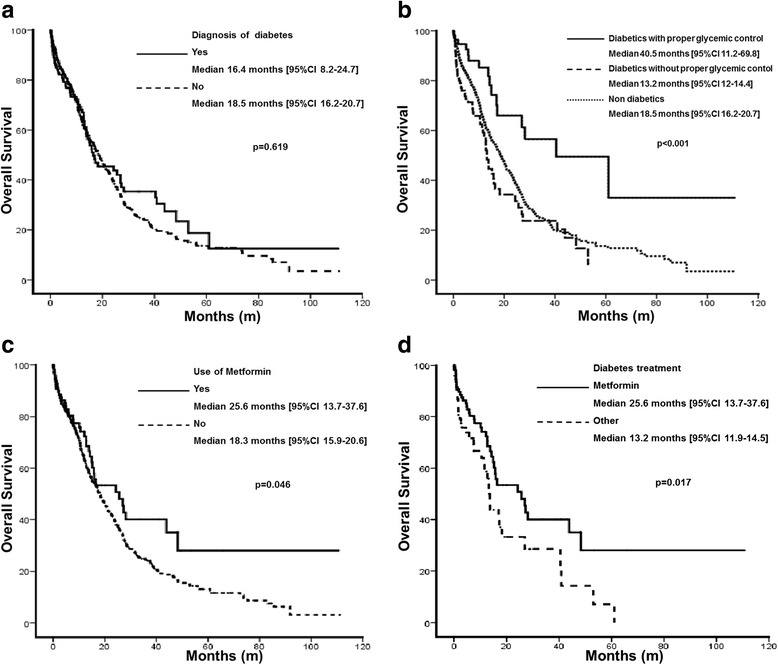
Overall survival comparisons of the patients. **a** Overall Survival comparison between diabetic and non-diabetic patients; **b** Overall Survival comparison between diabetic patients with and without proper glycemic control, and non-diabetic patients; **c** Overall Survival comparison between diabetic patients with and without use of metformin; **d** Overall Survival comparison between diabetic patients using metformin or using other antidiabetic treatment

The median follow-up time was 10.8 months (0.6-111.1), and the median OS for the entire cohort was 18.3 months [95 % CI 16.1-20.4]. The factors associated with OS are presented in Table 3. Female gender (*p* < 0.0001), no history of smoking (*p* < 0.01) and a lower stage at diagnosis (*p* < 0.0001) were associated with improved survival. Compared with the non-diabetic population, the patients with diabetes mellitus had a similar OS (18.5 vs. 16.4 months *p* = 0.619, Fig. 1a). However, the patients with proper glycemic control exhibited a better OS (40.5 months [95 % CI 11.2–69.8]) than did the diabetic patients without proper glycemic control (13.2 months [95 % CI 12–14.4]) and even exhibited a better OS than did the patients without diabetes mellitus (18.5 months [95 % CI 16.2–20.7]; Fig. 1b). Metformin use was associated with an improved OS in all of the patients, including the patients with and without diabetes mellitus (25.6 months [95 % CI 13.7–37.6] vs 18.3 months [95 % CI 15.9–20.6], *p* = 0.046; Fig. 1c). The factors that were independently associated with improved survival in the complete cohort are presented in Table 4.

**Table 3:** Overall survival and patient characteristics of the entire patient population (*n* = 1106) and the diabetic patient population (*n* = 186)

	Entire Patient Population (*n* = 1106)			Diabetic Patient Population (*n* = 186)		
Characteristic	OS (m) n	95 % CI	*p*	OS (m) n	95 % CI	*p*
Gender			<0.0001			0.087
Female	23.0 520	19.2–26.8		26.9 86	13.5–40.4	
Male	14.9 586	13–16.9		14.3 100	12–16.5	
Age			0.369			0.926
≤61	18.7 544	16.6–20.9		16.4 59	12.2–20.7	
> 61	17.4 562	12.8–22		18.3 127	7.3–29.2	
Smoking history			0.01			0.065
No	23.8 456	20.1–27.4		27.3 82	22.8–31.9	
Yes	15.2 650	13.4–17		13.8 104	11.8–15.9	
Histology			0.089			0.678
Other	14.2 352	11.1–17.2		14.9 60	10.3–19.6	
Adenocarcinoma	20.2 754	17.4–23		16.4 126	7.5–25.4	
Stage			<0.0001			0.012
II-IIIa	38.5 63	17.3–59.7		61.1 12	–	
IIIb-IV	17.2 1043	14.8–19.6		15.2 174	12.8–17.6	
Diabetes			0.619			
Yes	18.5 186	16.2–20.7				
No	16.4 920	8.3–24.5				
Metformin use			0.046			0.017
No	18.3 995	15.9–20.6		13.2 75	12.0–14.5	
Yes	25.6 111	13.7–37.6		25.6 111	13.7–37.6	
Insulin use			0.743			0.718
No	19 1087	16.4–20.4		17.0 165	8.4–25.6	
Yes	15.8 19	8.6–22.9		15.8 21	8.6–23.0	
Glibenclamide use			0.703			0.473
No	18.4 1046	16.4–20.5		17.0 126	5.2–28.7	
Yes	15.2 60	9.8–20.3		15.2 60	9.8–20.6	
Glycemic control			<0.001			<0.0001
Improper (≥130 mg/dL^a^)	12.1 208	10.1–14.1		13.2 127	12–14.4	
Proper (<130 mg/dL^a^)	19.8 887	17.2–22.3		40.5 58	11.1–69.8	
Glycemic control and diabetes^b^			<0.001			
Proper diabetic control	40.5 58	11.2–69.8				
Improper diabetic control	13.2 127	11.9–14.4				
Non-diabetic	18.4 920	16.2–20.8				

*OS* Overall Survival, *m* Months

^a^ Calculated mean serum glucose ^b^ One patient with missing glucose values

**Table 4:** Multivariate analysis of factors predicting survival in the entire patient population and in the diabetic patient population

Variable	All patients		
	HR	95 % CI	*P*
Gender	1.39	1.13–1.72	0.002
Smoking History	1.18	0.95–1.44	0.132
Histology	0.92	0.75–1.12	0.421
Clinical Stage IIIB-IV	2.03	1.33–3.08	0.001
ECOG Performance Status	1.26	1.10–1.43	0.001
Metformin use	0.53	0.37–0.75	<0.0001
Glycemic control	0.49	0.39–0.63	<0.0001
	Diabetic patient population		
	HR	95 % CI	*P*
Gender	1.36	0.80–2.33	0.256
Smoking History	1.35	0.79–2.31	0.277
Clinical Stage IIIB-IV	3.01	0.92–9.84	0.068
Metformin use	0.57	0.36–0.90	0.017
Proper glycemic control	0.40	0.22–0.71	0.002

### Factors associated with survival in the diabetic patients

The univariate analyses revealed that the factors associated with improved OS in the diabetic patients were lower stage at diagnosis, the use of metformin and proper glycemic control (Table 3). Compared with the diabetic patients who were using other antidiabetic medications, the patients taking metformin exhibited a significantly better OS (25.6 [95 % CI 13.7–37.6] vs 13.2 months [95 % CI 11.9–14.5]; *p* = 0.017, Fig. 1d). The largest improvement in OS was observed in the diabetic patients who achieved proper glycemic control (as defined by the calculated mean serum glucose; 40.5 vs 13.2 months; p <0.001). The diabetic patients with proper glycemic control exhibited a better OS than did both the poorly controlled diabetic patients and the non-diabetics (Fig. 1b). The multivariate analysis revealed that both the use of metformin (HR 0.57 [95 % CI 0.36–0.90]; *p* = 0.017) and proper glycemic control (HR 0.40 [0.22–071]; *p* = 0.002) remained significant protective factors in the diabetic patient population (Table 4).

## Discussion

Our results revealed that both the use of metformin and the maintenance of proper glycemic control are related to improved survival in the diabetic patients with advanced NSCLC. In our population, the use of metformin and the maintenance of proper glycemic control affected survival, independent of stage, histology and other antidiabetic treatments. Our results suggest that treatment with metformin, but not other hypoglycemic agents, is associated with a significant improvement in survival in patients with NSCLC and diabetes.

Evidence from epidemiological studies and meta-analyses has demonstrated associations of diabetes with several malignancies, including pancreatic [10], breast [11], prostate [12], colonic [13] and hepatocellular carcinomas [14]. In contrast, the relationship between diabetes and NSCLC is not well established, and epidemiological data have yielded conflicting results. Although some studies have reported an association between diabetes and NSCLC [15, 16], the majority has reported null or inverse relationships [17–19]. This potentially negative relationship is further illustrated by the fact that NSCLC has not been found to be associated with obesity, which is strongly related to the presence of diabetes [20]. Indeed, a high body mass index has been found to be a protective factor against NSCLC in some studies [21]. Further, a recently published meta-analysis by Zhang *et al.* concluded that metformin use in patients with diabetes appears to be associated with a reduced risk of lung cancer. This area requires further study and should be considered in the treatment of patients with diabetes and NSCLC [22].

The association between diabetes mellitus and survival in lung cancer patients is controversial [23] Whereas some studies suggest that patients with diabetes mellitus have worse prognoses due to comorbidities and disease complications that can be related to a reduced tolerance of treatment [24, 25], other studies have demonstrated that diabetic patients exhibited increased survival compared with non-diabetics [7, 26, 27]. The contradictory results between different studies might be the consequence of the analyses of heterogeneous populations. In the present study, we found that diabetes mellitus was not associated with an improved OS in all patients; however, our findings demonstrated that the diabetic patients with proper glycemic control exhibited a better OS than did the other diabetic patients and even the non-diabetics, which is consistent with previously published data [26].

Metformin has been explored as a pharmacological agent that may improve the survival of patients with several types of cancer. A recent meta-analysis reported associations between metformin and prolonged survival in patients with breast, colorectal, ovarian and endometrial cancers [28]. In NSCLC patients, the benefit of metformin on the prognosis has been demonstrated in large epidemiological studies [11] and in a meta-analysis [29]. Unfortunately, the main limitation of these studies is that they included heterogeneous patients and treatments and were thus prone to bias. In contrast, our report included a large set of NSCLC patients who were treated at a single reference institution where the prevalence of diabetes is high.

One potential explanation for the beneficial effects of metformin and proper glycemic control is that both are related to a reduction in circulating insulin levels. Insulin can stimulate both the insulin receptor (IR) and the insulin-like growth factor-1 receptor (IGF-1R), and both of these receptors are overexpressed in NSCLC patients and correlated with worse prognoses [30] and poor responses to targeted treatments [31]. Metformin has been demonstrated to interact with IGF signaling to reduce both the proliferation and migration of cancer cells [29]. There is a growing body of evidence regarding the effects of metformin on the multiple pathways that regulate carcinogenesis. Metformin inhibits mTOR translation initiation, the epithelial-mesenchymal transition (EMT), IL-6 secretion and STAT3 activity [32]. Thus, several preclinical experiments have tested combinations of metformin with radiotherapy [33], chemotherapy [34] and targeted therapies in lung cancer patients to overcome the mechanisms of resistance and to achieve synergy [35]. Metformin has been demonstrated to sensitize EGFR tyrosine kinase inhibitor (TKI)-resistant lung cancer cells by reversing the EMT and decreasing IL-6 signaling activation [32]. The combination of gefitinib (a reversible EGFR-TKI) and metformin induces a strong antiproliferative effect in NSCLC cell lines that harbor the wild-type LKB-1 gene [36]. Antiproliferative synergism between metformin and the multikinase inhibitor sorafenib via AMPK activation has also been demonstrated in NSCLC cells that harbor KRAS mutations [37]. Taken together, these data have led to the design of clinical trials utilizing combinations of metformin and targeted therapies for NSCLC patients who harbor specific mutations [38]. The ALMERA trial is an ongoing phase II trial conducted by the Ontario Clinical Oncology Group that will provide prospective evidence regarding the potential of metformin to improve the outcomes of standard cytotoxic therapy in locally advanced NSCLC patients [39].

Due to its retrospective nature, our study has some inherent limitations. HbA1c measurements were not available for the majority of the patients; therefore, we were unable to clearly identify patients with proper glycemic control. To resolve this issue, we calculated the mean plasma glucose levels using the fasting measurements from each patient. Although it was not ideal, this approach yielded the expected results in terms of the comparisons of the patient groups. Additionally, due to the retrospective nature of the data, we were unable to assess whether the patients who were not taking metformin had specific contraindications for its use or whether the patients with metformin prescriptions properly adhered to their treatment regimen. Furthermore, we had no data regarding whether patients who failed to achieve proper glycemic control were undergoing different types of treatment (e.g., chemotherapy rather than targeted therapy) that might have included high-dose steroids or had negative effects on proper adherence.

It is important to mention that our patients exhibited particularly prolonged average OS despite having advanced stage NSCLC. This finding may have resulted from the large incidence of EGFR-mutated tumors in our population [40, 41] and the broad access to clinical trials available at our institution. Some retrospective studies have associated the use of metformin with a lower risk of lung cancer, but the designs of these studies have been questioned due to time-related bias [42, 43]. To reduce the risk of this bias, both metformin use and proper glycemic control were assessed at the time of the lung cancer diagnoses.

It is possible that patients undergoing cytotoxic chemotherapy rather than targeted therapy would be more likely to stop taking metformin or that the drug could have different effects depending on the mutational statuses of the patients. However, the small number of EGFR and KRAS patients taking metformin made it difficult to draw any conclusions regarding these particularly interesting populations.

Finally, we were unable to gather data about the related metabolic comorbidities, such as waist circumference and lipid levels. However, given that mortality in this patient population is mostly cancer-related, we believe that the influences of such comorbidities on outcome would be minimal.

## Conclusion

The diabetic patients with proper glycemic control exhibited better OS than the diabetic patients without proper glycemic control and even exhibited better OS than did the patients without diabetes mellitus. Metformin use was independently associated with improved OS.

The use of metformin and the achievement of proper glycemic control have beneficial effects on the survival of patients with diabetes and advanced NSCLC. Prospective clinical trials of the use of metformin for lung cancer should be performed, particularly in selected populations in which the effects of metformin on signaling pathways could be potentially beneficial.

## Abbreviations

ADA, American Diabetes Association; AMPK, AMP-activated protein kinase; CI, confidence interval; EGFR, epidermal growth factor receptor; HbA1C, glycated hemoglobin; INCan, Instituto Nacional de Cancerología (Mexico City, Mexico); LKB1, liver kinase 1; NSCLC, Non-small cell lung cancer; OS, overall survival; SD, standard deviations

## Additional file

Additional file 1: Dataset supporting the conclusion of this article. (XLSX 115 kb)
